# Network Structure of Affective Communication and Shared Emotion in Teams

**DOI:** 10.3390/bs10100159

**Published:** 2020-10-17

**Authors:** Seung-Yoon Rhee, Hyewon Park, Jonghoon Bae

**Affiliations:** 1 Department of Business Administration, Hongik University, Seoul 04066, Korea; 2 Department of Economics, Finance, & Marketing, Tennessee Technological University, Cookeville, TN 38505, USA; hpark@tntech.edu; 3 Department of Business Administration, Seoul National University, Seoul 08826, Korea; jbae01@snu.ac.kr

**Keywords:** shared emotion, emotional contagion, affect, communication

## Abstract

This paper identifies the relative effectiveness of two mechanisms of emotional contagion on shared emotion in teams: explicit mechanism (active spreading of one’s emotion) and implicit mechanism (passive mimicry of others’ emotion). Using social network analysis, this paper analyzes affective communication networks involving or excluding a focal person in the process of emotional contagion by disaggregating team emotional contagion into individual acts of sending or receiving emotion-laden responses. Through an experiment with 38 pre-existing work teams, including undergraduate or MBA project teams and teams of student club or co-op officers, we found that the explicit emotional contagion mechanism was a more stable channel for emotional contagion than the implicit emotional contagion mechanism. Active participation in affective communication, measured by outdegree centrality in affective communication networks, was positively and significantly associated with emotional contagion with other members. In contrast, a team member’s passive observation of humor, measured by ego network density, led to emotional divergence when all other members engaged in humor communication. Our study sheds light on the micro-level process of emotional contagion. The individual-level process of emotional convergence varies with the relational pattern of affective networks, and emotion contagion in teams depends on the interplay of the active expresser and the passive spectator in affective networks.

## 1. Introduction

Emotion is contagious. Individuals tend to share emotion while interacting with others [1,2,3]. Shared emotion has been found within teams of community nurses and accountants [4], within professional cricket teams [5], between team leaders and followers [6,7], and between entrepreneurs’ video displays and their viewers [8]. Two distinct mechanisms are assumed to underlie the phenomenon of emotional contagion in teams. One is an explicit, deliberate process of emotional stories and related feelings [9,10]. The other is a rather implicit, automatic process whereby individuals passively mimic others’ emotional expression [11,12]. 

An explicit, deliberate process assumes that the more actively team members participate in affective communication, the more likely they will experience emotion similar to that of other team members [10]. In contrast, a rather implicit, automatic process assumes that without explicit exchange of emotion-laden stories and related feelings, team members can feel similar to each other by merely observing each other [13,14]. Individuals then passively mimic others’ emotional expression by attending to emotional cues through facial, postural, and behavioral expressions. 

Researchers assign different agency to a person in the course of emotional contagion, depending on the mechanism they choose. The explicit mechanism presumes that active participants in affective communication drive emotional contagion, whereas the implicit mechanism draws on passive spectators before emotional contagion proceeds. Although the explicit process is intentional, the implicit one is not necessarily so. Accordingly, recommendation by the explicit mechanism would be to induce each individual to exchange emotions actively with others, whereas by the implicit mechanism, it would be to develop a few energizers who shape group emotion sufficiently.

How many active members do we need to have emotional contagion in teams? The goal of this study is to identify the source of emotion contagion in teams, i.e., the relative effectiveness of the two mechanisms in the course of emotional contagion—explicit mechanism (active spreading of one’s emotion) and implicit mechanism (passive mimicry of others’ emotion). Unlike prior studies that concern the occurrence of emotional convergence at a group level (i.e., average team member’s emotion) [15], we seek to uncover each member’s contribution to emotional convergence at a group level. To this end, we opt for the social network perspective to disentangle the effects of the two emotional contagion mechanisms—active and passive participation in affective communication.

The social network perspective has been well recognized as a theoretical vehicle to unpack the structural constraints imposed on individual agency [16,17]. In particular, we combine the person-to-person exchange of affective communication with a network of affective communication. We then examine the relative effectiveness of affective communication involving or excluding a focal person, namely, ego. More specifically, we evaluate the role of the active expresser, measured by outdegree centrality in affective networks, in the sharing of emotions relative to that of the cohesive spectator, measured by ego network density.

We adopted the social network analysis method when analyzing experimental data from 38 pre-existing work teams. The participating work teams were comprised of undergraduate or MBA project teams and teams of student club or co-op officers recruited from a large Midwestern university in the United States. From the analysis, we found the following.

First, the explicit mechanism yields a stable and consistent impact on emotional contagion. Active participation in affective communication, measured by outdegree centrality in affective communication networks, is positively and significantly associated with emotional contagion, reversely measured by the difference in positive emotion between ego and his or her team. A few energizers do matter. Second, the implicit mechanism generates mixed effects on emotional contagion. For the exchange of humor, passive observation, measured by ego network density, leads to emotional divergence from team shared emotion. In contrast, the same passive observation strengthens emotional contagion when ego’s partners exchange affective communication that involves agreement. These findings suggest that intervention by a few emotional energizers may backfire when the remaining spectators passively respond and when the content of communication is directed to humor among members.

## 2. Theory and Hypotheses

### 2.1. Mechanisms of Shared Emotion in Teams

Emotions are social [9,18]. We express emotion in social interaction, and interaction partners may observe our expressed emotions and be influenced by them. Shared emotion is, thus, an emergent psychological property of teams that arises through member interactions [1,2,4,15]. 

To better understand the process of emotional contagion in teams, researchers have theorized two different mechanisms of emotional contagion. One of the mechanisms involves an explicit, deliberate process of emotional contagion through exchanges of emotional stories and related feelings [9,19]. Social sharing of emotion “occurs in discourse, when individuals communicate openly with one or more persons about the circumstances of the emotion-eliciting event and about their own feelings and emotional reactions” [10] (p. 65). Being exposed to a sender’s emotional stories and expression can facilitate the receiver’s experience of similar emotion, which likely results in empathy and a feeling of emotional communion [10,20]. 

The other mechanism of emotional contagion is a more implicit, automatic process whereby individuals unintentionally mimic others’ emotional expression [11]. The notion of primitive emotional contagion explains the implicit process, such as “the tendency to automatically mimic and synchronize facial expressions, vocalizations, postures and movements with those of another person and consequently to converge emotionally” [13] (pp. 153–154). Individuals acquire emotional cues by observing others’ facial, postural, and behavioral expressions [2], which facilitates the implicit, automatic process of emotional contagion [21]. Other implicit, subconscious processes include vicarious learning, behavioral entrainment, and interaction synchrony [12,22,23,24]. 

Both explicit and implicit emotional contagion mechanisms suggest that the length and frequency of affective interactions among individuals can influence the extent to which they share emotion [25]. More specifically, these mechanisms suggest that the longer and the more frequently team members participate in affective interactions or observe each other’s emotional expression, the more likely that they will feel similar and thus result in shared emotion or emotional convergence. 

Several antecedents of shared emotion corroborate the idea of the length and frequency of affective interactions leading to shared emotion. Membership stability and task and social dependence, which indicate frequent and long-term interactions among team members, have been found to enhance the likelihood that the team experiences shared emotion [2]. Similarity of affect depends on the presence of work ties, and similarity in affective responses tends to be more pronounced among employees who occupy similar positions in the social network structure [26]. These studies underscore the importance of the number of interactions, that is, the more interactions team members have with each other, the more similar their emotion will become. The underlying assumption of these findings is that the teams or organizational social structures can shape the length and frequency of social interactions, which, in turn, influences the extent to which members can disseminate and acquire emotional information.

While the logic of length and frequency of social interactions influencing emotion sharing is most appealing at a team level, it becomes less plausible when considered at an individual level. The vast literature on team dynamics suggests that individual team members differ from each other in terms of their level of participation in team processes and their social status in the team [27]. It has also been noted that the extent to which each individual member transfers emotions through interpersonal relations may vary depending on his or her affective disposition, emotional intelligence, and social relationships [12]. Therefore, rather than assuming that all team members will participate in social interactions in the team with equal length and frequency, it would be more convincing to argue that each team member’s social interaction will vary in nature.

Indeed, the two emotional contagion mechanisms suggest that individuals engage in different types of interactions in the course of emotional contagion, one indicating an active participation in affective communication and the other indicating a rather passive form of participation through mere observation of others’ affective expression. Accordingly, individual team members’ degree of emotional convergence toward team shared emotion will depend on the individuals’ active or passive participation in affective communication with other members.

Moreover, individual members’ emotional assimilation with others may vary with the nature of their participation, i.e., the individuals’ active or passive participation in affective communication with other members. For example, as long as an individual experiences arousal in the course of active affective communication, he or she may attribute such arousal to the experience of positive emotion. If this is the case, active participation in affective communication is more likely than passive participation to underlie the experience of positive emotion. In a similar vein, the passive spectator of affective communication among others may feel isolated from the others, thus experiencing a decline in positive emotion. 

Our attempt to parse out the effects of the two emotional contagion mechanisms aims to compare the relative effectiveness of the two mechanisms in facilitating individual team members’ emotional convergence toward team shared emotion. In doing so, we pay attention to minority emotion or emotion that does not necessarily follow the team’s average emotion by accounting for individual-level variance in the degree of emotional convergence.

### 2.2. Affective Communication and Sharing of Emotion

We address the differing role of affective interactions, either active or passive, in the course of emotional sharing. We define affective communication as a spoken exchange of thoughts, opinions and feelings, which has the potential to arouse emotion in individuals engaged in the exchange. In this study, we use affective interaction and affective communication interchangeably. Affective communication has been found to strengthen interpersonal relationships [10,28] more so than the sharing of facts and information [29]. 

Individuals who participate actively in social interactions with other team members tend to have more opportunities to express their ideas, opinions, and emotions. Research on affective communication indicated that emotionally expressive individuals were more likely to infect others with their emotion [30,31,32]. Also, the narrator of emotional stories and her audience tended to develop a stronger social bond when audiences responded with enthusiasm [33]. Researchers have often used the idea of an active participant in affective communication influencing others’ emotion in experimental settings, where a confederate expresses emotion to induce similar emotion in the experiment’s participants [34,35,36]. 

Those who are emotionally expressive may shape others’ emotion not only because they have more expression opportunities but also because, in doing so, others perceive them as powerful. In a study of emotional convergence between roommates and dating partners over time, partners with more power have been found to influence the emotion of the less powerful partner [37]. Also, lower status partners reported a wider change in their level of emotion than those with higher status, for emotional convergence to occur. Power is likely to be associated with individuals who occupy a central position in affective communication network than with those in peripheral positions, due to the dominant position the central individuals take in team processes.

These findings suggest a flow of emotion from active participants of affective communication to other team members. Thus, the gap between emotions of an individual team member and those of other team members will become narrower, especially when the individual actively engages in affective communication. Therefore, we hypothesize that individuals holding a central position in an affective communication network will exhibit a convergence of positive emotion toward the team’s shared emotion.

**Hypothesis** **H1.** *As a focal person’s active participation in affective communication increases, the difference in the level of positive emotion between the person and his or her team declines*.

Another avenue through which emotional contagion occurs is the implicit, subconscious process of mimicking others’ emotion. Past research has demonstrated that individuals can assimilate others’ emotion through mere observation of their facial expressions, vocal tone, and body language [2,21]. It has also been proposed that an agent’s emotion might affect not only the person at whom the emotion was directed, but also the third parties who simply observed the agent’s emotion [38]. 

In particular, observers have been reported to detect more overt movements such as rapid vocal pace and hearty laughter with higher accuracy, due to the salience of those movements [2]. In fact, our notion of passive participation in affective communication includes observation of both subtle and overt non-verbal, as well as verbal, emotional expressions. This inclusive nature of passive participation in affective communication heightens the likelihood that an individual team member, as a bystander or a spectator of others’ affective communication, will be able to detect and be influenced by others’ emotion. Hence, we hypothesize that an individual team member’s emotion will converge toward team shared emotion even when engaged passively in affective communication.

**Hypothesis** **H2.** *As affective communication excluding a focal person increases, the difference in the level of positive emotion between the person and his or her team declines*.

The basic assumption behind the discussion above is that interactions in teams are not symmetric. Some are more active than others. In a similar vein, the sharing of emotions in teams is not symmetric [39]. Some are more expressive and responsive than others. Besides individual differences in affective states, one important and yet largely neglected candidate for such asymmetry is the pattern of affective communication in teams, i.e., affective networks, which cannot be relegated into relational qualities between two parties such as intimacy of contacts.

A network model of social influence in this regard would suggest two distinct patterns, which vary in the disproportionate role of influential or opinionated leaders [40,41,42]. A model of differentiated interactions views those who are central to interactions as a source of social contagion [43,44], whereas one of undifferentiated interactions focuses on the clustering of interactions, which underlie mutual reinforcement among persons and thus illicit social contagion [45,46]. To the extent that affect is linked to attitude, a network model of social influence is applicable to the sharing of emotions in teams. The two mechanisms of contagion mentioned above, i.e., deliberate and automatic processes, correspond to the models of differentiated and undifferentiated interactions. H1 posits that the active expresser, i.e., an influential person, serves as a major source of emotion contagion in teams. In comparison, H2 suggests that cohesive spectators would underlie the sharing of emotions in teams.

Indeed, network scholars found the role of interaction patterns in the sharing of moods, such as happiness and loneliness, among close contacts that last for a long period of time [47,48]. The active role of the influencer in shaping moods in teams was also found in the authority relation, such as the interaction of a formal leader and followers [49]. Increasing attention is also given to affective contagion on online platforms such as Facebook, a form of computer-mediated communication, which is integral to our everyday interactions [50,51,52].

Against this backdrop, we opt for a social network analysis and examine the short-term process of emotion contagion in teams. The analysis of affective networks allows us to disaggregate emotion contagion in a team into individual acts of sending or receiving affects. In doing so, we seek to identify who contributes most to emotion contagion, namely, the role of the active expresser vis-à-vis the passive spectator in a cohesive team.

## 3. Methods

### 3.1. Participants and Procedure

To test the hypotheses, we conducted an experiment with 38 pre-existing work teams, each consisting of three to six members. Among the participating teams, the majority were undergraduate project teams (32 teams), and the rest included three MBA project teams, two teams of student club officers and members, and a team of co-op officers. We recruited the teams through newspaper advertisements, emails, fliers around campus, and courses at a large Midwestern university. Five experimenters independently conducted the experiment for a period of over four months. 

Upon arrival, each participating team was randomly assigned to one of the two experimental conditions: joy and neutral emotion conditions. There were 68 participants in 19 teams in the joy condition and 77 participants in 19 teams in the neutral emotion condition. The emotion manipulation procedure was part of a related study. To minimize the effect of experimental conditions on the current study, we combined data from the joy and neutral emotion conditions and used the combined dataset for the hypotheses test. Later, we checked for any experimental condition effect and found no significant effect on our dependent variable, i.e., the degree of emotional convergence. Hence, we dropped the experimental condition variable from the analysis from then on.

Team members were first asked to fill out a questionnaire that assessed how they felt at the moment (Time 1 emotion). Next, they worked on a team task known as “space survival,” often used to teach the topic of team decision-making in organizational behavior courses [53]. Each team was asked to rank ten items (i.e., rope, pistols, oxygen tanks, etc.) according to their importance for the 200-mile trip on the Moon and to come up with as many creative and feasible ways to use each of the ten items. Information about conditions on the Moon, including air, temperature, and gravity, was provided. Each team had thirty minutes to work on the task. While team members worked on the team task, their verbal and nonverbal interactions were video recorded. 

After the team task, team members completed a questionnaire that assessed their current positive emotion (Time 2 emotion). The questionnaire also included items measuring team satisfaction and individual learning for a related study. Teams were debriefed at the end.

*Ethical considerations.* All subjects gave their informed consent for inclusion before they participated in the study. The study was conducted in accordance with the Declaration of Helsinki, and the protocol was approved by the Health Sciences and Behavioral Sciences Institutional Review Board at the University of Michigan (B03-00003881-R1).

### 3.2. Team Member Interactions (Process Analysis)

Video-recorded member interactions were coded to capture the affective communication network within teams. Coding video-recorded member interactions is a relatively unobtrusive and objective method to capture various types of team member interactions including verbal communication as well as nonverbal behaviors such as laughter. Three independent raters coded the video-recorded team member interactions for a period of six months. 10 of the 38 videotapes were coded by all three raters; the others were coded by each of the three raters independently, once the rate of agreement among all three raters reached over 90%. Inter-coder reliability score was used to measure the extent to which independent coders agreed on the coding of team member interactions applying the same coding scheme. The three raters discussed and agreed on the coding scheme in Table 1 before coding the 38 videotapes. The inter-coder agreement rate of over 90% indicates that the interpretation of the content can be regarded as fairly objective [54].

We particularly focused on the affective communication with positive nature and chose two types of affective communication—active agreement and humor—as the focus of our analysis. We followed Bales’ categorization of positive social-emotional communication [55,56] and focused on the two types of interactions that were described as the most positive in nature. The first type of interaction is related to showing team solidarity by raising others’ status, and the second is related to tension release by using jokes or laughs. We chose active agreement and humor as exemplars of the two types of positive social-emotional communication. ‘Active agreement’ refers to accepting and supporting other team members’ ideas by responding with a relatively high level of arousal and energy (e.g., “So you want to make a hot air balloon. That would work!”; “I like that idea!”). It is likely to raise the social status of the person who initially suggested the idea and strengthen the emotional bond among the team members. ‘Humor’ category refers to inducing laughter by using humor [57]. For a control purpose, we included negative interactions in our analysis, which refers to disagreement and rejection of other team members’ ideas in a straightforward manner (e.g., “I don’t think that’s important.”) [58].

The unit of analysis was a ‘thought unit’ [59] or an ‘action unit’ uttered or displayed by a person. A thought unit is a “sequence of a few words conveying a single thought” [60] (p. 559), and an action unit indicates a single action taken by a person. For example, if a person said, “we are doing great!” and clapped, it was counted as two different incidents of affective communication.

### 3.3. Measurement

#### 3.3.1. Positive Emotion

We used seven items to measure the positive emotion of joy at an individual level. Joy belongs to a team of emotions that is positive in valence and high in arousal [61]. It shares the same conceptual field with gladness, happiness, elation, and exhilaration [62,63]. A hierarchical cluster analysis of emotions [64] showed emotions of delight and excitement belonging to the same category as the joy emotion. The question read: “Please tell us to what extent you feel this way right now, that is, at the present moment.” A 7-point scale was used: 0 = ‘not at all’, 6 = ‘extremely’. Cronbach alpha was 0.94. After finishing the team task, team members rated their current positive emotion, using the same items as above (Cronbach alpha = 0.95).

#### 3.3.2. Affective Communication Network

The raters counted the frequencies of positive and negative interactions, respectively, and summarized them in the form of matrices of affective networks. In particular, rows (*i*) in the matrix represented senders giving positive or negative responses, and columns (*j*) represented receivers of the responses. Values in each of the cells (*a*(*i*,*j*)) indicated frequencies of positive or negative responses *i* sent to *j*. The matrix, therefore, depicts how frequently affective communication occurred among team members. For the computation of affective communication network measures, we transformed the matrices into the binary ones, such that each entry whose frequency of affective interactions was above the sample median was set to one and otherwise set to zero. From this procedure, we constructed two types of affective communication networks, one for active agreement and the other for humor.

For the test of hypotheses, we used UCINET VI software [65] and derived two network measures from the above-mentioned affective communication networks: a person’s outdegree centrality and his or her ego network density. First, outdegree centrality is the number of team members to whom a person sends affective communication with a frequency above the sample median. For example, a person’s outdegree centrality in the affective communication network of active agreement is two when he or she sends active agreement to two team members and his or her frequency of doing so is above the sample median. Outdegree centrality concerns the extent to which a person actively participates in affective communication, such as active agreement and humor. 

Second, unlike group-level density, the ego network density indicates the presence or absence of interactions among ego’s direct partners. Accordingly, ego’s affective interactions, per se, are not included in the computation of ego network density [66]. Hence, ego network density captures the extent to which a person’s partners exchange affective communications among themselves. Ego network density increases as an increasing number of ego’s partners join affective communication. Hence, the higher the ego density, the more opportunities the ego has to observe other members’ exchanges of affective communication. Formally, it is defined as:

$\frac{\sum {}_{i}\sum {}_{j}b(i,j)}{N(N-1)}$ where b(i,j)=1 if a(i,j)>M and M is the median frequency of affective interaction of a certain type, and N is number of team members with whom a person exchanges affective communications and who are indexed by *i* and *j*.

#### 3.3.3. Control Variables

A demographic variable (participants’ gender) and a personality variable (trait promotion and prevention focus) were entered into the analyses to control for their potential effects on the process of emotional contagion. Negative ties outdegree centrality was entered as well to control for its potential effect on emotional convergence.

### 3.4. Model Specification

For the test of our hypotheses on emotional convergence, we relate emotional divergence to a set of covariates, including the properties of affective communications. For person *i* in group *j*, the model is specified as follows:

(1) $$\log(Y_{ij})=Χ\beta +g_{j}+u_{ij}$$

where the dependent variable is the absolute difference in the level of positive emotion between person *i* and his or her group *j*; *X* is a vector of observed covariates; $g_{j}$ is a time-invariant, group-specific unobserved factor; and $u_{ij}$ is the remaining error term, which is independently distributed across *i* and *j*.

We chose a log linear specification to mitigate the influence of extreme values in the dependent variable. Due to dependence on multiple observations for each team, we allowed for correlations in repeated measures from the dependent variable and adjusted the standard error estimates of parameters in Equation (1). To this end, we assumed a simple random effects model for the correlation of repeated measures and fit the model to the data using GENMOD algorithm by SAS. The estimates we obtained were equivalent to the generalized least squares [67,68]. White’s robust standard errors were used to test the hypotheses [69]. Pairwise correlations among covariates were below 0.4, indicating that multicollinearity may not have been substantial in estimation.

## 4. Results

Descriptive statistics and the correlation matrix are reported in Table 2. Table 3 gives the coefficient estimates, including robust standard errors. We also estimated models with a random coefficient for outdegree centrality and found that the results were consistent with those reported in Table 3. The unit of analysis is a person in a given experimental group. We identified two different types of affective communication and examined their respective effects on the degree of emotional convergence. The number of observations varied from 110 to 121 because we excluded cases where the ego network density was not computable. That is, the ego network density was not mathematically defined for the individuals who have zero or one partner in the network of affective communication. Four controls were included in the models: gender, trait promotion focus, trait prevention focus and negative ties outdegree centrality, all of which were measured at an individual level. The estimated coefficients for these control variables remain consistent across two different types of affective communications, i.e., active agreement and humor. Gender effect was marginally significant, whereas personality had significant effects on emotional convergence. Individuals with trait promotion focus exhibited emotional divergence, while those with trait prevention focus reported emotional convergence. Individuals’ negative ties outdegree centrality was associated with emotional divergence.

We identified the two distinct channels of emotional contagion by evaluating the following two network variables: a person’s outdegree centrality and his or her ego network density. The coefficient for outdegree centrality reflects the extent to which one’s own affective communication induces one’s emotion to converge into team shared emotion. In comparison, the coefficient for ego network density indicates the extent to which affective communication not involving ego leads one’s emotion to be similar to team shared emotion. 

Note that the negative coefficient indicates a reduction in emotional divergence, which is an increase in emotional convergence. Two patterns merit attention. First, outdegree centrality in both humor (β = −0.060, *p* < 0.05) and agreement (β = −0.056, *p* < 0.05) is negatively associated with emotional divergence. The negative effects of outdegree centrality suggest that a person actively involved in affective communication is likely to have personal emotion similar to team shared emotion, thus supporting Hypothesis H1.

Next, ego network density is positively associated with emotional divergence, thus rejecting Hypothesis H2. The positive effects of ego network density cast doubt on the second channel of emotional contagion. Although the insignificant effect of agreement ego density precludes any statistical inference (β = 0.013, n.s.), and the effect of humor ego density strongly suggests that affective communication of humor excluding a person may hinder emotional contagion (β = 0.138, *p* < 0.05). Rather, a person passively observing his or her partners’ affective communication of humor tends to develop emotion that is remote from the team’s shared emotion. 

We undertook additional analysis to see whether the passive spectator was immune to team shared emotion. To this end, we replaced the variable of outdegree centrality with that of indegree centrality. Indegree centrality indicates the number of affective communications the focal person receives from other team members with a frequency above the sample median. Note that the pairwise correlation between indegree and outdegree centralities is 0.195 for humor communication and 0.313 for agreement communication, respectively. This suggests that the two measures are relatively distinct in data. The estimated coefficients for humor and agreement communication are as follows. 

For humor communication:

Log(Y) = 0.361 + 0.009 × indegree centrality + 0.077 × ego network density −0.091 × gender + 0.075 × promotion focus −0.049 × prevention focus + 0.059 × negative ties outdegree. 

For agreement communication:

Log(Y) = 0.312 + 0.022 × indegree centrality + 0.006 × ego network density −0.112 × gender + 0.091 × promotion focus −0.039 × prevention focus + 0.040 × negative ties outdegree. 

As is shown above, indegree centrality is positively associated with emotional divergence, but in a statistically insignificant way for both humor and agreement communication. The *p*-value for humor indegree centrality is 0.360, and the *p*-value for agreement indegree centrality is 0.254. These patterns imply that, at least, active involvement in affective communication (outdegree centrality) is a better predictor of emotional contagion than reception of affective communication (indegree centrality) in our experiment.

To further examine the workings of ego network density, we tested the presence of a curvilinear relationship between ego network density and emotional divergence. Table 4 reports the results. The squared term for ego network density was positive in the case of humor (β = 0.622, *p* < 0.01) but negative in the case of agreement (β = −0.482, *p* < 0.05). This implies that, in the case of humor, the passive observation of the others’ affective communication leads a person’s emotion to diverge increasingly from team shared emotion. In contrast, emotional contagion is strengthened as ego density in agreement communication increases. 

**Figure 1:**
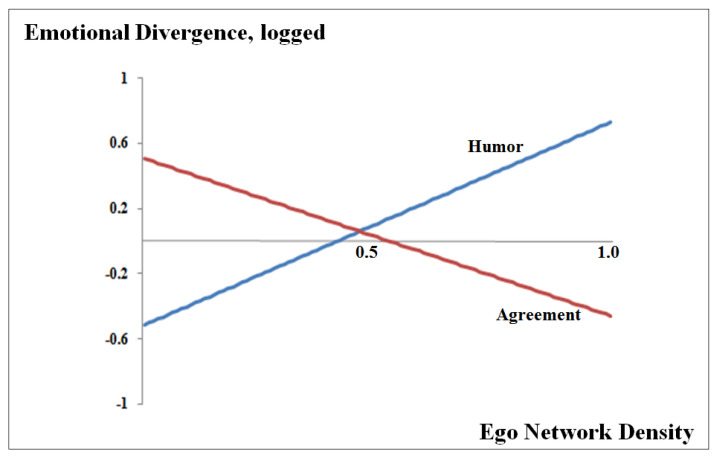
Effect of ego network density on emotional divergence. Note. The three graphs are derived from the partial derivatives of the estimated Equation (1) with respect to ego network density. The optimal ego network densities for humor and agreement are 0.414 and 0.526, respectively.

Figure 1 illustrates the two opposing patterns in the working of ego network density. When the ego network density of humor is above 0.5, a person’s emotion diverges rapidly from his or her group emotion. In contrast, a person imitates team shared emotion easily as the ego network density of agreement becomes more than 0.5. A possible explanation for the different effects of the two different types of ego network density would be that agreement and humor communication differ in their nature. According to Bales’ description of the two types of interactions, active agreement reflects team solidarity or members’ discretionary efforts to solidify the team [55]. On the other hand, humor interaction is considered a positive way of releasing tension, the purpose of which is a bit remote from building social bonds and team cohesiveness. We suspect that the inherently social nature of active agreement may facilitate the emotional contagion process even for the mere observer of the interaction.

## 5. Discussion and Conclusions

In this study, we addressed the relative effectiveness of affective communication involving or excluding a focal person in the course of emotional contagion within teams. From the experiment with 38 pre-existing work teams, we found that the explicit emotional contagion mechanism (i.e., active affective communication by a person) was a more stable channel for emotional contagion than the implicit mechanism (i.e., affective communication passively observed by a person). For example, active communication of affect, measured by outdegree centrality in affective network, was positively and significantly associated with emotional contagion, reversely measured by the difference in positive emotion between ego and his or her team. When team emotion converged via positive interactions, individual members’ emotion turned out to be more positive, especially when individuals were sending out this interaction frequently. In contrast, the passive observation of humor, measured by ego network density, led to emotional divergence when all the partners of ego engaged in humor communication. 

Theoretically, this study contributes to the literature on emotional contagion and team emotion by looking deeper into the individual-level process of emotional convergence toward team shared emotion. In doing so, we suggest that the implicit and explicit mechanisms of emotional contagion work in a different manner and that group intervention by a few emotional energizers may backfire in some contexts. The extant research mostly presumes that subconscious processes (e.g., primitive emotional contagion) and conscious processes (e.g., emotion-laden conversation) equally impact the emergence of collective emotion [19]. Insofar as these two processes perform in different ways, the success of the contagion process should vary substantially depending on how the two processes interact with each other. As with prior studies [39], one implication of our study is that the relative importance of the two processes will vary with situation-specific motives, including the motivation to feel strong emotions in the workplace, which may determine the final state of emotional similarity (i.e., divergence or convergence) in teams. 

Moreover, this study shows how structural patterns of affective communication shape individual and team shared emotion, thus linking emotion and social network studies. The extent to which each team member transfers emotions may vary depending on his or her affective traits, emotional intelligence, and social relationships [12]. Neglecting these differences would mask two theoretically distinct and practically important processes of emotional contagion. One is contagion driven by all or the majority of members in a team, whose individual emotions are equally important in determining the shared emotion of the team. The other is a process driven mainly by a few members in the team. In this case, the presence of a few active senders or receivers in communication may determine the success of the contagion process. The notion of affective communication networks can provide rich information about team dynamics and their causal relationships with team outcomes. In particular, with the measures of network density and degree centrality, we show the possibility of disentangling the effects of implicit and explicit mechanisms for collective emotion in teams. 

Three theoretical implications merit further discussion along this line. First, we seek to identify who contributes to emotion contagion in teams. When a team consists of only two people, the analysis of dyadic interactions will suffice. However, for a team of more than two people, dyadic analysis would be problematic, provided that the pairwise exchange of emotions depends on the context in which a given exchange of emotions is embedded. A network analysis of affective interactions is one way to consider the contextual dependence. Accordingly, we suggest that the individual-level process of emotional convergence varies with the relational pattern of affective.

Second, we evaluate changes in affective states in the context of short-term interactions. Relational approach to emotional contagion concerns the sharing of moods among close contacts that last for a long period of time [47,48]. Given the paucity of research on emotion contagion, our study adds additional support to the role of affective networks in the genesis of emotions [70,71]. To this end, we measured the individual acts of expressing and responding to affective states, a process that traced the short-term emotional dynamics in teams and that differed substantially from the measure of name-generators aimed at capturing stable, long-term relationships [72].

Third, we show that emotion contagion in teams depends on the interplay of the active expresser and the passive spectator in affective networks. The interplay varies with the type of emotion in question, such as humor and agreement. More importantly, the interplay unveils the limiting role of influential or opinionated leaders. Consistent with prior findings [42,73], our study shows that the passive spectator sets a limit to the influence of expressers in everyday encounters. For example, repetitive exposure to expressive acts may underlie emotion contagion when team members are evenly involved in humor communication. In contrast, a few influential leaders who dominate humor communication may hinder the remaining spectators to share similar affective states. The effects are revered for agreement communication, which resembles political discourse in the sense that spectators are easily affected by a few influential leaders.

This research opens new avenues for future research. First, emotional contagion research has assumed that individuals interacting with each other tend to share similar emotions. However, it is necessary to distinguish between the following two cases: one where emotional similarity leads to social interactions and the other where social interactions lead to emotional similarity. This is because the first case may pose a threat to the importance of interpersonal interactions in the contagion process. Without controlling for such endogeneity in relations formation, it is difficult to vindicate the model of emotional contagion [74].

Second, there has been mounting evidence that shared emotion influences team performance, but the findings have been inconclusive in terms of whether positive, negative, or mixed emotion improves team creativity or decision quality [34,75,76,77]. Our findings suggest that there is an individual-level variance in terms of the extent to which the individual’s emotion converges toward or diverges from the team’s shared emotion. By looking deeper into the different emotional contagion processes and resulting variance in the level of emotional convergence, we may unlock the black box of how collective emotional processes influence team performance.

Third, researchers have assumed that socially induced variations on individual emotions tend to converge into shared emotion in teams. Social interactions may lead to changes in individual members’ emotions [78], yet the team as a whole may fail to develop shared emotion in certain cases. For example, two individuals of incompatible emotions, such as excitement and grief, may not develop shared emotion even after social interactions. Rather, they are likely to stick to their initial emotion even after being exposed to the other’s dissimilar emotion. Hence, it is important to identify boundary conditions under which social interactions lead to shared emotion in teams.

One possible boundary condition would be power and status differences among team members. High-power individuals tend to show high arousal emotions (e.g., excitement or anger), while low-power individuals mostly respond with low arousal ones (e.g., contentment or guilt) [79]. Team gender composition can be considered as a potential moderator based on the finding that female members tended to be more susceptible to leader emotional influence than males did [80]. Strength of team identity and the level of identification with the team may impact emotional contagion process as well. A strong single team identity may facilitate emotional contagion among the members, whereas multiple team identities and weak identification with the team may result in divergent emotion in reaction to team events [81,82,83]. Future research may delve into the role of such social structural factors.

Finally, there is a growing interest in the emotional contagion process in non-face-to-face contexts such as virtual teams or telecommuting contexts [84,85,86]. Although individuals may mimic each other’s affective expressions online, using electronic media may make it more difficult than in a face-to-face interaction context because of content ambiguity and the limited non-verbal cues [87]. Negative emotion can be more easily transmitted electronically than positive emotion because negative expressions violate societal norms and thus become more noticeable and contagious. By examining how the virtual context, a relatively new work setting, impacts the emotional contagion process, we may extend our understanding of the affective dynamics of work teams.

In summary, the current literature on shared emotion has shown limited understanding of the individual-level emotional contagion process. The emotional contagion process is more than simply reacting in an emotionally similar way [88] but rather involves building upon and shaping each other’s emotion. We attempted to address this concern by focusing on the different emotional contagion mechanisms and their relative effectiveness in the emotional contagion process, thus shedding light on the micro-level processes of emotional contagion and their influences on collective emotion.

## Figures and Tables

**Table 1:** Coding scheme for affective communication.

Components	Description	Examples
Active agreement	Acceptance of and support for other members’ ideas. Needs certain level of arousal. Affirming with low level of arousal or affirmation intended to fill space does not count.	“I like that!” “That might not be such a bad idea!” “That’s a great idea!”
Humor	Making of friendly jokes Trying to amuse or entertain Includes positive responses to joking, such as smiling, giggling, or laughing	A: “Cool, use pistols to shoot crater for dust storm.” B: “Shoot ourselves.” Team: (laughter) C: “If we don’t have spacesuits, we’re dead anyways.” D: “We’re lunar exploration crew, we have to have space suits.” E: “I know, like you don’t go scuba diving in jeans and sweatshirt.” Team: (laughter)

**Table 2:** Descriptive statistics and correlations.

Variables		Mean	SD	1	2	3	4	5	6	7
1	Humor outdegree centrality	1.39	0.99							
2	Humor ego density	216.34	363.84	−0.240						
3	Agreement outdegree centrality	1.49	0.97	0.215	−0.096					
4	Agreement ego density	275.33	405.22	−0.111	0.194	−0.453				
5	Gender	0.59	0.49	0.077	0.027	0.066	−0.051			
6	Promotion focus	3.85	0.82	0.179	−0.026	0.017	0.034	0.143		
7	Prevention focus	3.44	1.04	−0.071	0.221	−0.036	0.098	0.039	0.322	
8	Negative ties outdegree centrality	1.11	1.02	0.128	−0.168	0.132	0.023	0.116	−0.004	−0.003

Note: Correlations greater than ∣0.168∣ are significant at *p* < 0.05.

**Table 3:** Emotional Divergence and Affective Network.

	Model 1	Model 2
Constant	0.400 ** (0.169)	0.388 * (0.201)
Humor outdegree centrality [H1]	−0.060 * (0.027)	
Humor ego density [H2]	0.138 * (0.082)	
Agreement outdegree centrality [H1]		−0.056 * (0.034)
Agreement ego density [H2]		0.013 (0.099)
Gender	−0.084 † (0.060)	−0.099 † (0.062)
Promotion focus	0.091 ** (0.034)	0.095 ** (0.034)
Prevention focus	−0.059 * (0.029)	−0.033 (0.029)
Negative ties outdegree centrality	0.065 * (0.036)	0.049 (0.041)
Number of observations	121	110
Log likelihood	−33.17	−33.46

Note: † *p* < 0.10; * *p* < 0.05; ** *p* < 0.01 (one-tailed test); robust standard errors are in parentheses. Generalized least squares equivalent estimates are reported.

**Table 4:** The non-linear effects of ego network density on emotional divergence.

	Model 1	Model 2
Constant	0.441 ** (0.158)	0.351 * (0.207)
Humor outdegree centrality [H1]	−0.044 * (0.027)	
Humor ego density [H2]	−0.515 * (0.289)	
Humor ego density, squared [H2]	0.622 ** (0.261)	
Agreement outdegree centrality [H1]		−0.079 * (0.034)
Agreement ego density [H2]		0.507 † (0.334)
Agreement ego density, squared [H2]		−0.482 * (0.283)
Gender	−0.089 † (0.058)	−0.087 † (0.064)
Promotion focus	0.103 ** (0.034)	0.097 ** (0.035)
Prevention focus	−0.064 * (0.028)	−0.031 (0.030)
Negative ties outdegree centrality	0.072 * (0.035)	0.039 (0.040)
Number of observations	121	110
Log likelihood	−29.69	−32.09

Note: † *p* < 0.10; * *p* < 0.05; ** *p* < 0.01 (one-tailed test); robust standard errors are in parentheses. Generalized least squares equivalent estimates are reported.
